# Scaling Drug Clearance from Adults to the Young Children for Drugs Undergoing Hepatic Metabolism: A Simulation Study to Search for the Simplest Scaling Method

**DOI:** 10.1208/s12248-019-0295-0

**Published:** 2019-03-08

**Authors:** E. A. M. Calvier, E. H. J. Krekels, T. N. Johnson, A. Rostami-Hodjegan, D. Tibboel, Catherijne A. J. Knibbe

**Affiliations:** 1 Division Systems Biomedicine and Pharmacology, Leiden Academic Centre for Drug Research (LACDR), Leiden University, Leiden, The Netherlands; 2 Certara UK Limited, Sheffield, UK; 3 Centre for Applied Pharmacokinetic Research (CAPKR), University of Manchester, Manchester, UK; 4 Intensive Care and Department of Pediatric Surgery, Erasmus University Medical Center—Sophia Children’s Hospital, Rotterdam, The Netherlands; 5 Department of Clinical Pharmacy, St. Antonius Hospital, P.O. Box 2500, 3430 EM Nieuwegein, The Netherlands

**Keywords:** maturation, paediatrics, PBPK modelling, physiologically based pharmacokinetics, prediction

## Abstract

Previous research showed that scaling drug clearance from adults to children based on body weight alone is not accurate for all hepatically cleared drugs in very young children. This study systematically assesses the accuracy of scaling methods that, in addition to body weight, also take age-based variables into account for drugs undergoing hepatic metabolism in children younger than five years, namely scaling with (1) a body weight-based function using an age-dependent exponent (ADE) and (2) a body weight-based function with fixed exponent of 0.75 (AS0.75) combined with isoenzyme maturation functions (MF_PBPK_) similar to those implemented in physiologically based pharmacokinetic (PBPK) models (AS0.75 + MF_PBPK_). A PBPK-based simulation workflow was used, including hypothetical drugs with a wide range of properties and metabolized by different isoenzymes. Adult clearance values were scaled to seven typical children between one day and four years. Prediction errors of ± 50% were considered reasonably accurate. Isoenzyme maturation was found to be an important driver of changes in hepatic metabolic clearance in children younger than five years, which prevents the systematic accuracy of ADE scaling. AS0.75 + MF_PBPK_, when accounting for maturation of isoenzymes and microsomal protein per gram of liver (MPPGL), can reasonably accurately scale hepatic metabolic clearance for all low and intermediate extraction ratio drugs except for drugs binding to alpha-1-acid glycoprotein in neonates. As differences in the impact of changes in system-specific parameters on drugs with different properties yield differences in clearance ontogeny, it is unlikely that for the remaining drugs, scaling methods that do not take drug properties into account will be systematically accurate.

**Electronic supplementary material:**

The online version of this article (10.1208/s12248-019-0295-0) contains supplementary material, which is available to authorized users.

## Introduction

Accurate scaling of drug plasma clearance (CLp) from adults to children is important for the definition of first in child doses and hence robust study design involving younger children. To date, physiologically based pharmacokinetic (PBPK) models represent the most mechanistic method to scale CLp across the paediatric age range. PBPK models quantify the interactions between drug-specific and system-specific parameters and predict paediatric CLp by accounting for developmental changes in the system-specific parameters and how they impact drugs with specific properties. Application of these models is considered best practice in pharmaceutical industry, but obtaining PBPK ontogeny functions for a given drug is time-consuming and complex due to the requirement of a wide range of drug-specific and system-specific information. Moreover, all information may not always be available for each drug or each population. This leads to a need for simplified scaling functions which are more convenient for defining paediatric CLp in pharmacometrics. As multiple system-specific parameters may change in the paediatric population and as the impact of each of these changes on paediatric CLp may be different for each given drug with different characteristics, the challenge in developing simplified scaling functions is to aggregate all relevant information in functions with a limited number of scaling variables. Various simplified clearance scaling methods for the paediatric population have been proposed. Allometric scaling using a fixed exponent of 0.75 (AS0.75) is one of the simplest scaling methods, as it only uses body weight as scaling variable. However, AS0.75 has been shown to lead to large over-predictions of hepatic metabolic CLp in children younger than 5 years, especially when isoenzymes are immature (1,2).

As scaling based on body weight alone was found not to lead to systematic accurate scaling, other proposed scaling functions that rely on the use of additional age-based variables are of interest. Mahmood *et al.* have proposed the age-dependent exponent method (ADE) that was found to outperform AS0.75 in young children (1,3). ADE relies on the use of an allometric equation with exponents of 1.1, 1.0 and 0.9 for ages 0 (term neonates)–3 months, >3 months–2 years and > 2–5 years, respectively, for all drugs as most recently reported (3). While this method is claimed to be applicable to any drug irrespective of their elimination route, this method does not account for the differences in isoenzyme maturation, which are known to impact hepatic metabolic clearance and to vary greatly (4).

Another proposed scaling method uses AS0.75 together with isoenzyme maturation functions that are similar to those implemented in PBPK models (AS0.75 + MF_PBPK_) (4). In addition to body weight, this method also requires information on the fraction of the drug metabolized by each isoenzyme in adults, as well as on isoenzyme maturation. This method does not explicitly account for maturation in haematocrit and plasma protein abundance. Based on data of five CYP3A substrates, AS0.75 + MF_PBPK_ was found to be accurate in children older than 3 months, but it could lead to inaccurate predictions in younger children for some drugs (4).

While ADE and AS0.75 + MF_PBPK_ represent potentially viable options to accurately scale clearance in children under five years of age (1,3,4), no systematic investigation of their accuracy has been undertaken. The aim of this study was therefore to systematically assess the accuracy of paediatric CLp scaling with ADE and AS0.75 + MF_PBPK_ in children younger than five years for drugs undergoing hepatic metabolism that are not substrates for transporters, to identify drug properties that are predictive for accurate scaling with these methods. This will ultimately allow for the *a priori* assessment of the suitability of these scaling methods for different paediatric ages and for drugs with known properties, by defining the minimum level of complexity that is required for accurate CLp scaling. For this, a previously developed PBPK-based simulation workflow was used (5). In this workflow, hypothetical drugs that are substrates for common hepatic isoenzymes are generated, covering the entire potential drug parameter space regarding plasma protein binding, blood-to-plasma partitioning and intrinsic microsomal clearance. PBPK modelling principles are used to obtain ‘true’ hepatic metabolic CLp values for all hypothetical drugs in adults and children of various ages. Subsequently, CLp values scaled from ‘true’ adult values to paediatric values with ADE and AS0.75 + MF_PBPK_ are compared to ‘true’ CLp values in children, and drug properties that lead to systematically accurate scaling in various ages are identified.

## Methodology

A PBPK-based simulation workflow was used (5) that was running in R (a software environment for statistical computing and graphics) version 3.3.1 with R studio interface version 0.99.902 (6). In this workflow, ‘true’ adult and paediatric hepatic metabolic CLp values for hypothetical drugs with a wide range of properties that are substrates for known hepatic enzymes were generated using PBPK-based simulations, based on the dispersion model for hepatic metabolic CLp (7,8). This model was selected as it has been reported to better predict CLp than the well-stirred model for drugs with a high extraction ratio, while both models lead to equivalent CLp prediction for other drugs (7,9). Subsequently, the accuracy of scaling the ‘true’ adult CLp values to paediatric CLp values with the two scaling methods was assessed, by comparing CLp values scaled by ADE and AS0.75 + MF_PBPK_ to ‘true’ paediatric CLp values.

### PBPK Simulation Workflow

#### Hypothetical drugs

A total of 84,000 hypothetical drugs were generated, with all possible combinations of values for the following three drug-specific variables:

*Plasma protein binding*. The hypothetical drugs were assumed to exclusively bind to either human serum albumin (HSA) or alpha-1 acid glycoprotein (AAG). The unbound drug fraction in plasma (fu) in adults ranged from 1 to 100%, with 8 equidistant intermediate values. Equations by Rodgers and Rowland (10) were used to derive the affinity to plasma proteins from the fu and the concentration of the binding plasma proteins in adults (11). The affinity to plasma proteins was assumed to remain constant with age.

*Blood-to-plasma partition coefficient (Kp)*. Kp values of 0.35 and 0.8 and values from 1 to 40 with 38 intermediate equidistant values were selected, reflecting different extents of drug diffusion into the red blood cells (12,13). Kp was assumed not to change with age.

*Total unbound intrinsic clearance value of one microgram of liver microsomes (total CLint,mic)*. Total CLint,mic ranged between 0.56·10^−6^ and 0.209·10^−3^ mL min^−1^ μg^−1^ microsomal protein in adults (14), with 98 equidistant intermediate values. These different values reflect difference in both affinities for and abundances of isoenzymes.

#### System-Specific Variables

The accuracy assessment of two scaling methods was performed in seven typical paediatric individuals, including term neonates of one and fifteen days, infants of one month, six months, and one year, and children of two and four years. CLps were scaled from a typical twenty-five-year-old adult. The demographic and system-specific parameters of the PBPK model for these typical individuals can be found in Appendix 1.

For each investigated paediatric age, isoenzyme maturation (CLint,mic maturation) was implemented as a near continuous variable. To do so, first, a realistic range of isoenzyme maturation values was defined for each age by taking the maximum and minimum isoenzyme maturation values reported for 15 isoenzymes. A minimum limit of 5% isoenzyme maturation was set. For all isoenzymes but SULT1A1, isoenzyme maturation values were taken from the Simcyp® library. For SULT1A1, maturity was taken to have been reached at birth (15). Then, intermediate values across these ranges were taken with 1% increments, to allow for the investigation of CLp maturation of drugs metabolized to different extents by all possible combinations of multiple isoenzymes, an important feature since most drugs are metabolized by several isoenzymes.

### Computations

#### Step 1: ‘True’ CLp

For each hypothetical drug, ‘true’ hepatic metabolic CLp values were generated for the typical adult and children as described previously. More details can be found in Appendix 1.

For each paediatric age, ‘true’ relative paediatric CLps were computed as in Eq. (1), reflecting ‘true’ paediatric CLp as a percentage of ‘true’ adult CLp:

1 $$ {}^{`}{true}^{'} relative\ paediatric\  CLp=\frac{{}^{`}{\mathrm{true}}^{'}\ \mathrm{paediatric}\  CLp}{{}^{`}{\mathrm{true}}^{'}\ \mathrm{adult}\  CLp}\times 100 $$

#### Step 2: CLp Scaling

First, for each hypothetical drug and for each of the different percentages of isoenzyme maturation defined for each age, the ‘true’ adult hepatic metabolic CLp values from step 1 were scaled to each typical paediatric individual using ADE and AS0.75 + MF_PBPK_ scaling functions according to Eqs. (2) and (3), respectively.

2 $$ \mathrm{ADE}-\mathrm{based}\ \mathrm{paediatric}\ \mathrm{CLp}={}^{`}{\mathrm{true}}^{'}\mathrm{adult}\ \mathrm{CLp}\times {\left(\frac{\mathrm{BWpaediatric}}{\mathrm{BWadult}}\right)}^{ADE} $$

3 $$ \mathrm{AS}0.75+{\mathrm{MF}}_{\mathrm{PBPK}}-\mathrm{based}\ \mathrm{paediatric}\ \mathrm{CLp}={}^{`}{\mathrm{true}}^{'}\mathrm{adult}\ \mathrm{CLp}\times {\left(\frac{\mathrm{BWpaediatric}}{\mathrm{BWadult}}\right)}^{0.75}\times {\mathrm{MF}}_{\mathrm{PBPK}} $$

In these equations, BW stands for body weight, ADE equals 1.1, 1.0 and 0.9, for ages 0 (term neonate)–3 months, > 3 months–2 years, and > 2–5 years, respectively (3) and MF_PBPK_ corresponds to the different percentages of isoenzyme maturation defined for each age, as also used in the PBPK model for the generation of ‘true’ relative paediatric CLps (see Appendix 1).

In literature, there are two different interpretations of MF_PBPK_ in use and both were investigated in this work. MF_PBPK_ was either expressed as percentage of adult unbound intrinsic clearance per gram of liver (MF_PBPK-liver_), which accounts for maturation in both isoenzyme activity and microsomal protein per gram of liver (MPPGL). Alternatively, MF_PBPK_ was expressed as percentage of adult unbound intrinsic clearance per microgram of microsomes (MF_PBPK-microsomes_), which only accounts for maturation of isoenzyme activity. Therefore, for MF_PBPK-liver_, maturation in MPPGL as implemented in the PBPK model for the generation of ‘true’ relative paediatric CLps was also used.

For comparative purposes, Eq. (4) was used to calculate the exponent that in the allometric equation of the ADE method would yield perfect scaling of ‘true’ adult hepatic metabolic CLp to ‘true’ paediatric hepatic metabolic CLp; this will be referred to as ‘true’ allometric exponent.

4 $$ {}^{`}{\mathrm{true}}^{'}\  EXP=\frac{\log 10\left({}^{`}\mathrm{t}{\mathrm{rue}}^{'}\ \mathrm{relative}\ \mathrm{paediatric}\ \mathrm{CLp}\right)}{\log 10\left(\frac{\mathrm{BWpaediatric}}{\mathrm{BWadult}}\right)} $$

#### Step 3: Assessment of CLp Scaling Accuracy

For each drug and each percentage of isoenzyme maturation in each paediatric age, the accuracy for both ADE and AS0.75 + MF_PBPK_-based CLp scaling was numerically assessed using the prediction error (PE). PE was computed for each ‘true’ paediatric hepatic metabolic CLp generated in step 1 and its corresponding scaled value in step 2 using Eq. (5).

5 $$ \mathrm{PE}\ \left(\%\right)=\frac{\mathrm{scaled}\ \mathrm{CLp}-{}^{`}{\mathrm{true}}^{'}\ \mathrm{paediatric}\ \mathrm{CLp}\ }{{}^{`}{\mathrm{true}}^{'}\ \mathrm{paediatric}\ \mathrm{CLp}}\times 100 $$

For each paediatric age and investigated percentage of isoenzyme maturation, the scaling performance of both methods was visually assessed in plots of ‘true’ and scaled relative paediatric CLp values. The scaling accuracies were also compared to scaling accuracy of AS0.75. AS0.75 CLp predictions were computed as in Eq. (2), with an exponent of 0.75 in all ages. Analogue to previous systematic assessments of simplified scaling methods, accurate CLp scaling was defined as scaled values having a PE within ± 30% (2,5,16).

#### Step 4: Drug Properties Predictive for Accurate Scaling

To define scenarios in which each scaling method systematically yields accurate paediatric hepatic metabolic CLp values, the combined impact of plasma protein binding to HSA or AAG and diffusion in red blood cells was assessed using the following categorization:

- drugs influenced neither by plasma protein maturation (fu = 1) nor by haematocrit maturation (Kp = 1) (*n* = 100);
- all hypothetical drugs binding to HSA, including drugs with fu = 1 (*n* = 42,000);
- all hypothetical drugs binding to AAG, including drugs with fu = 1 (*n* = 42,000).

These categories were, then, further subcategorized based on the extraction ratio in adults (ER) as having either a low (ER ≤ 0.3, *n* = 19,002), intermediate (0.3 < ER ≤ 0.7, *n* = 17,684), or high (ER > 0.7, *n* = 5314) ER in adults.

## Results

### CLp Scaling Accuracy

Table I provides the range of ‘true’ relative paediatric hepatic metabolic CLp values for each age, as well as the corresponding range of PE obtained when scaling hepatic metabolic CLp with ADE, AS0.75 + MF_PBPK-liver_ and AS0.75 + MF_PBPK-microsomes_. For comparative purposes, PE values upon AS0.75 scaling are provided as well (Table I).

**Table I:** Assessment of Paediatric CLp Scaling Accuracy, Expressed as Prediction Error, for Different Ages, and ‘True’ Relative Paediatric CLp

Age	‘True’ relative paediatric CLp^*a*^ (range) (%)	Prediction error (range)			
		ADE^*b*^ (%)	AS0.75 + MF_PBPK_liver_^*c*^ (%)	AS0.75 + MF_PBPK_microsomes_^*d*^ (%)	AS0.75^*e*^ (%)
One day	0.26–13.31	− 74–1224	− 87–23	− 79–92	− 24–3745
Fifteen days	0.29–8.14	− 54–1220	− 87–20	− 81–87	31–3645
One month	0.32–9.15	− 51–1305	− 87–22	− 80–89	31–3679
Six months	1.09–15.56	− 33–853	− 85–19	− 77–82	18–1578
One year	1.62–21.26	− 36–739	− 84–22	− 76–83	5–1281
Two years	2.67–29.3	− 42–536	− 83–22	− 75–77	− 10–890
Four years	5.07–43.02	− 37–437	− 80–33	− 72–82	− 21–567

^*a*^Paediatric CLp expressed as percentage of adult value

^*b*^Age-dependent exponent

^*c*^Scaling using AS0.75 in combination with a maturation function expressed in percentage of adult unbound intrinsic clearance per gram of liver

^*d*^Scaling using AS0.75 in combination with a maturation function expressed in percentage of adult unbound intrinsic clearance per microgram of microsomes

^*e*^Allometric scaling using a fixed exponent of 0.75

ADE, AS0.75 + MF_PBPK_liver_ and AS0.75 + MF_PBPK_microsomes_ capture changes in ‘true’ hepatic metabolic CLp for part of the hypothetical drugs, as can be seen from the PE ranges which all include ± 30% in each age for each of these scaling methods. However, each of these methods also leads to inaccurate paediatric CLp predictions for other hypothetical drugs in each age. Scaling with ADE, AS0.75 + MF_PBPK_liver_ and AS0.75 + MF_PBPK_microsomes_ yields extreme PE values that, on an absolute scale, are at least 437, 80, or 77%, respectively, with higher values for younger ages (Table I). As maturation in system-specific parameters may impact drugs with different properties differently and since scaling based on ADE or AS0.75 + MF_PBPK_ does not account for drug properties, these methods are not able to capture the wide range in ‘true’ CLp values and, therefore, yield a wide range of PEs in the different ages. Unlike AS0.75 + MF_PBPK_, ADE does not account for differences in isoenzyme maturation, which translates to a wider PE range with this scaling method. However, compared to the use of AS0.75, ADE does yield a range of PEs that is greatly reduced.

### Impact of Isoenzyme Maturation on CLp Scaling Accuracy

**Fig. 1:**
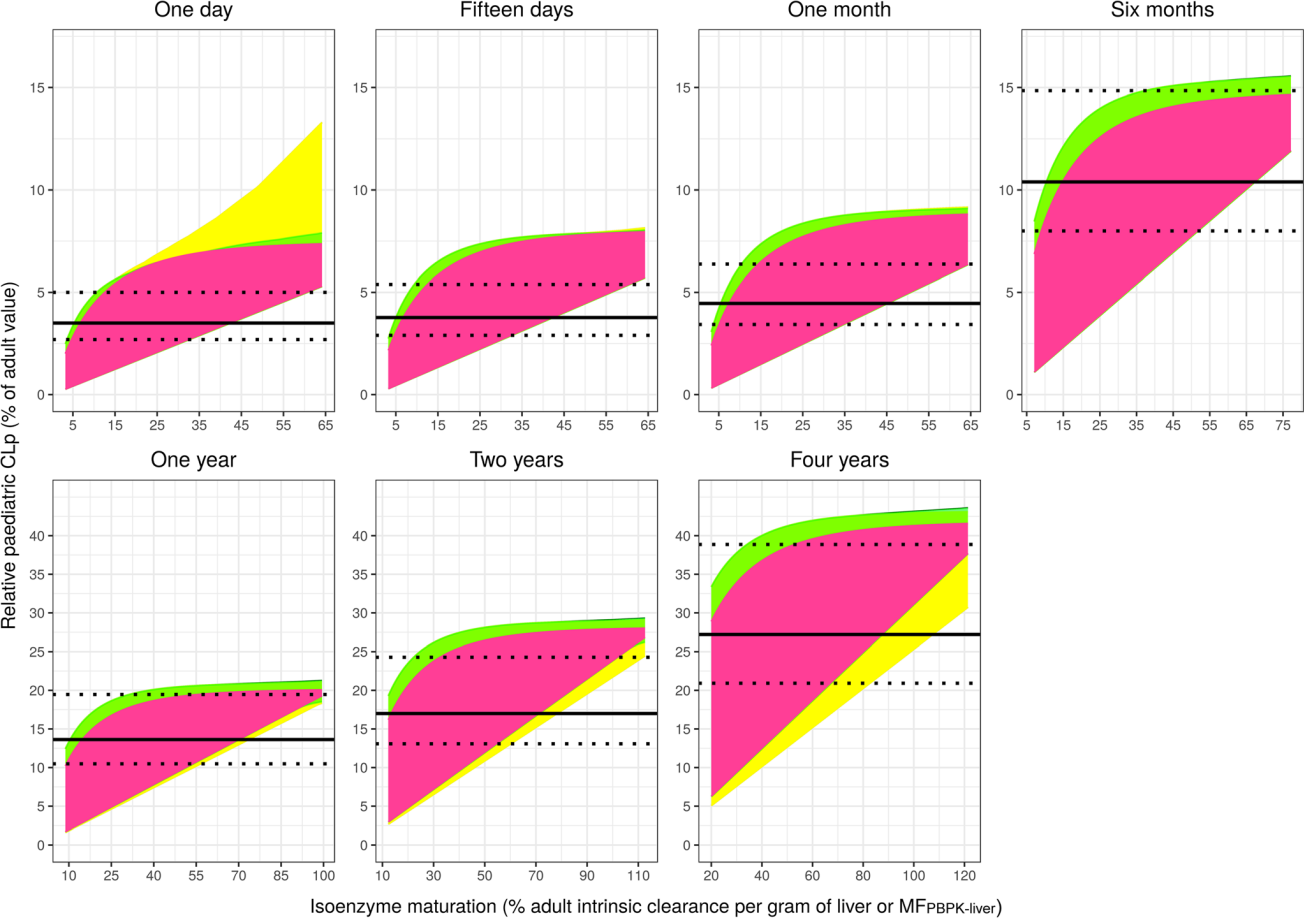
Relative paediatric CLp (% of adult value) obtained with ADE scaling (solid black line with ± 30% PE as dotted black lines) and ‘true’ relative paediatric CLp (pink, green or yellow areas) for all hypothetical drugs *versus* the respective isoenzyme maturation range in the studied typical paediatric individuals. Different colours represent hypothetical drugs with different properties, with pink representing drugs not binding to plasma proteins (fu = 1) that are also in equilibrium between plasma and red blood cells (Kp = 1). Green and yellow are used to depict drugs that diffuse into red blood cells to different extents and that bind to HSA or AAG, respectively, to different extents (including fu = 1). Under the pink area, the pink, yellow and green areas overlap completely; therefore, the combination of pink and green areas shows the results for all drugs binding to HSA and the combination of pink, green and yellow areas shows the results for drugs binding to AAG. Note that the scales on the x- and y-axes may be different for different ages

**Fig. 2:**
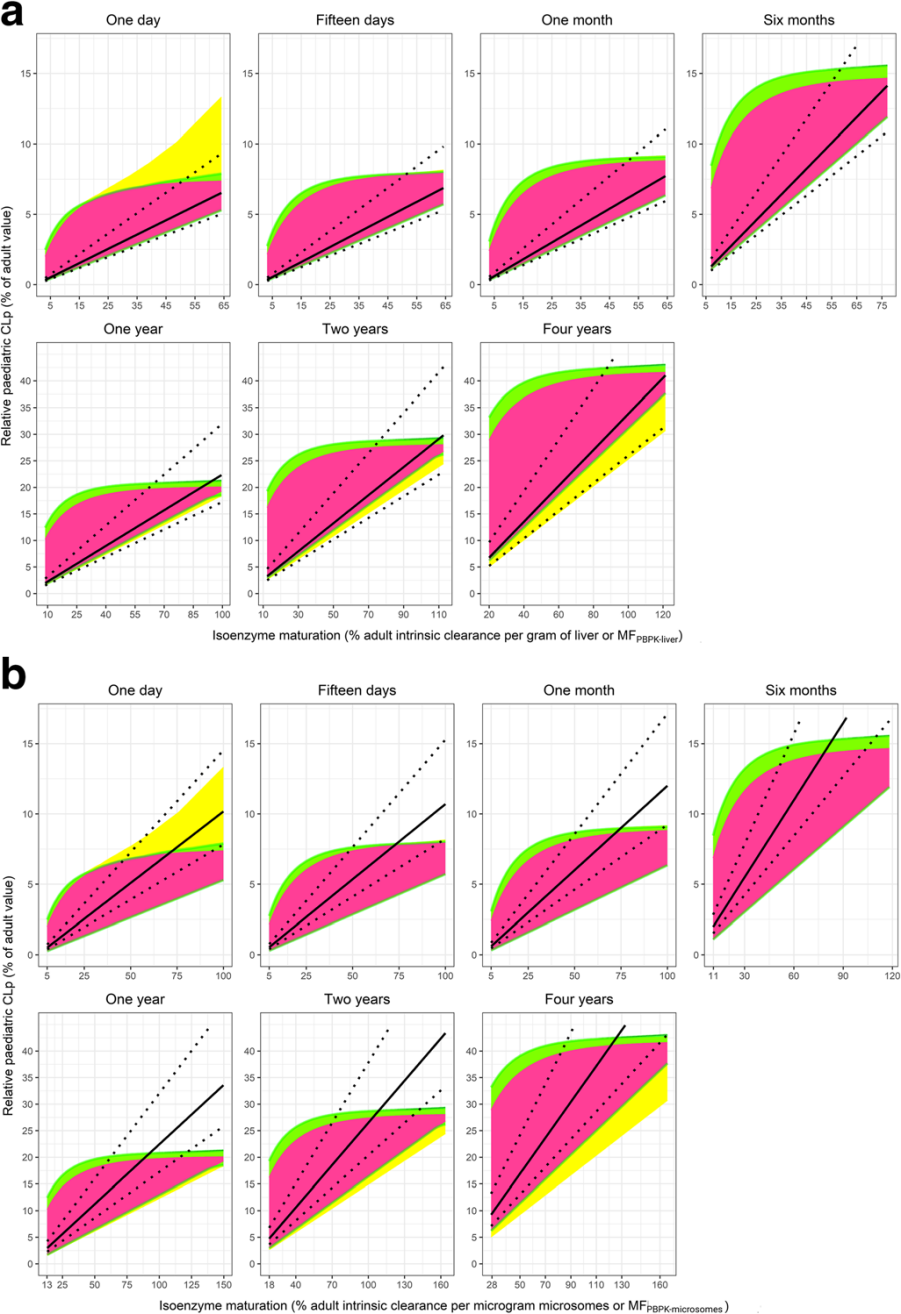
Relative paediatric CLp (% of adult value) obtained with AS0.75 + MF_PBPK-liver_ scaling (**a**) and AS0.75 + MF_PBPK-microsomes_ scaling (**b**) (solid black line with ± 30% PE as dotted black lines) and ‘true’ relative paediatric CLp (pink, green and yellow areas) for all hypothetical drugs *versus* the respective isoenzyme maturation range in the studied typical paediatric individuals. Different colours represent hypothetical drugs with different properties, with pink representing drugs not binding to plasma proteins (fu = 1) that are also in equilibrium between plasma and red blood cells (Kp = 1). Green and yellow are used to depict drugs that diffuse into red blood cells to different extents and that bind to HSA or AAG, respectively, to different extents (including fu = 1). Under the pink area, the pink, yellow and green areas overlap completely; therefore, the combination of pink and green areas shows the results for all drugs binding to HSA and the combination of pink, green and yellow areas shows the results for drugs binding to AAG. Note that the scales on the x- and y-axes may be different for different ages

Figures 1 and 2 compare the scaled relative paediatric hepatic metabolic CLp with a ± 30% PE using, respectively, ADE or AS0.75 + MF_PBPK_ *versus* the ‘true’ relative paediatric hepatic metabolic CLp for all hypothetical drugs in each investigated paediatric age and across their respective isoenzyme maturation range. The x-axis in Figs. 1 and 2a displays isoenzyme maturation per gram of liver (MF_PBPK_liver_) which reflects both MF_PBPK_microsomes_ and maturation in MPPGL, while the x-axis in Fig. 2b displays isoenzyme maturation per microgram of microsomes (MF_PBPK_microsomes_).

Figure 1 shows that while ADE can accurately scale hepatic metabolic CLp for some hypothetical drugs and for some isoenzyme maturations in each age, this scaling method can lead to a wide range of PEs due to the large variation in ‘true’ relative paediatric CLp values. Figure 1 also shows that for each typical paediatric individual, ‘true’ CLp values that are lower and higher than those predicted with ADE and a ± 30% PE range are found, with over-predictions for the lowest isoenzyme maturation values and under-predictions for highest isoenzyme maturation values.

Figure 2a shows that AS0.75 + MF_PBPK-liver_ does not generally lead to over-prediction of hepatic metabolic CLp in the studied age range, but under-predictions may occur, especially when isoenzyme maturation is low. When enzyme maturation in this approach is expressed relative to adult intrinsic activity per microgram of microsomes (AS0.75 + MF_PBPK-microsomes_), both over- and under-predictions of paediatric CLp for different drugs are observed in all ages (Fig. 2b).

**Fig. 3:**
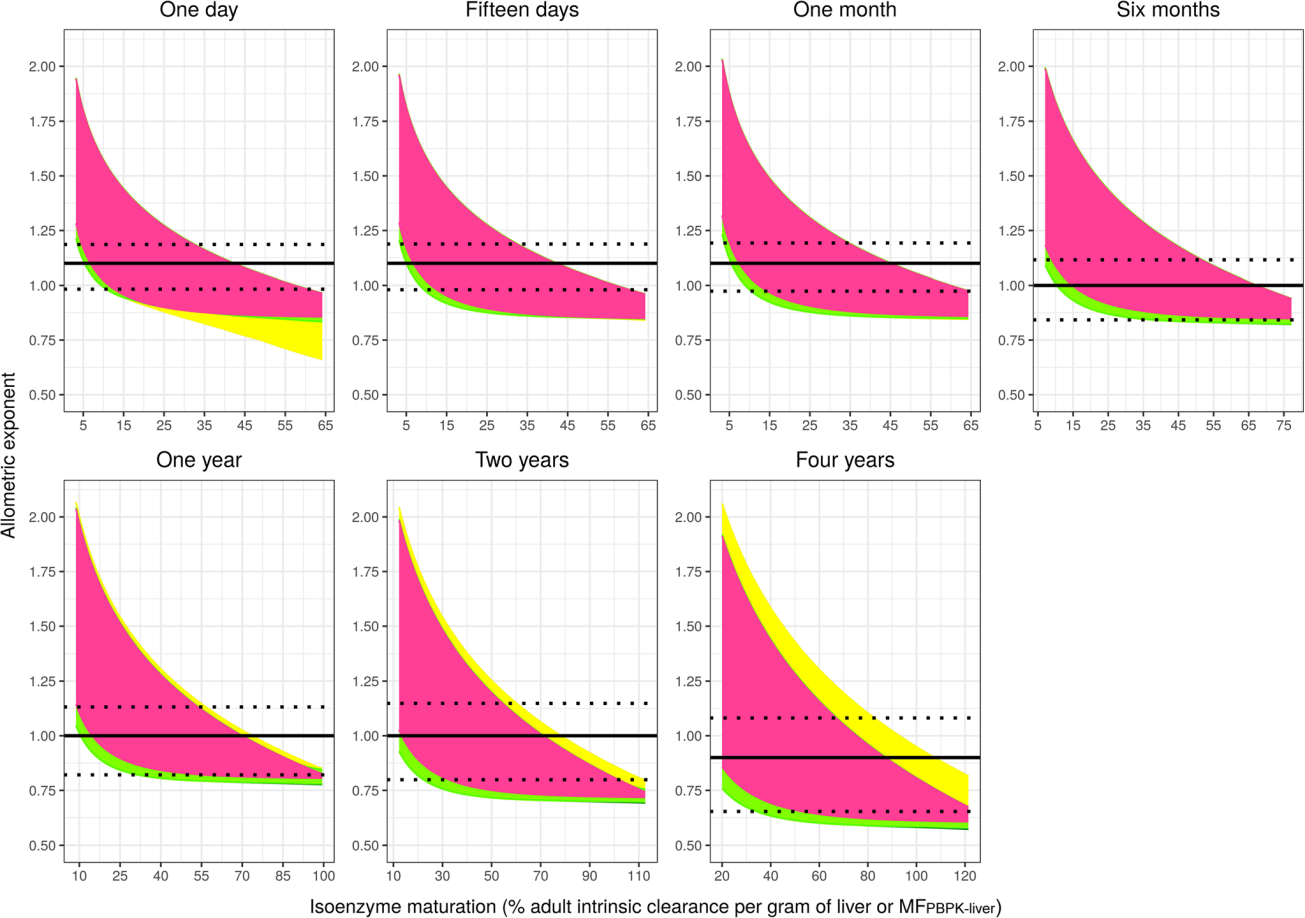
‘True’ allometric exponent (pink, green and yellow areas) and ADE exponent used to scale CLp (solid black line with ± 30% PE in CLp as dotted black lines) for all hypothetical drugs *versus* the respective isoenzyme maturation range in the studied typical paediatric individuals. Different colours represent hypothetical drugs with different properties, with pink representing drugs not binding to plasma proteins (fu = 1) that are also in equilibrium between plasma and red blood cells (Kp = 1). Green and yellow are used to depict drugs that diffuse into red blood cells to different extents and that bind to HSA or AAG, respectively, to different extents (including fu = 1). Under the pink area, the pink, yellow and green areas overlap completely; therefore, the combination of pink and green areas shows the results for all drugs binding to HSA and the combination of pink, green and yellow areas shows the results for drugs binding to AAG. Note that the scale on the x-axes may be different for different ages

It was determined for all hypothetical drugs, what the ‘true’ allometric exponent would be if it was estimated in the typical paediatric patients within the respective isoenzyme maturation range. Figure 3 illustrates how the range of ‘true’ allometric exponents compares to the allometric exponent used in ADE scaling. High values of ‘true’ relative paediatric CLp will yield low values of ‘true’ allometric exponent, and, therefore, the reverse trends with isoenzyme maturation can be observed in Fig. 3 as compared to Fig. 1. The ‘true’ allometric exponent varies considerably within each paediatric age, ranging from 0.57 to 2.07 across all ages. Table I and Fig. 3 show that changing the allometric exponent in the scaling function with age, as proposed with ADE scaling, will lead to an overall improved scaling for more hypothetical drugs, but it also illustrates that there is no single exponent that will accurately scale hepatic metabolic CLp for all drugs in each age.

### Identification of Drug Properties Predictive for Accurate CLp Scaling

In Figs. 1 and 2, results were grouped in 3 categories in order to assess the combined impact of drug binding to HSA or AAG and drug diffusion in red blood cells on ‘true’ relative paediatric hepatic metabolic CLp. This categorization does not explain the observed variability in ‘true’ relative paediatric CLp values, which can be seen by the observed values for each category outside the ± 30% PE range of the scaling methods. As such, drug binding to HSA or AAG and drug diffusion in red blood cells do not allow for the definition of drug variables for which ADE or AS0.75 + MF_PBPK_ systematically leads to accurate scaling.

Further categorization of these results based on the ER of drugs in adults was not found to allow for the definition of drug variables for which ADE systematically leads to accurate hepatic metabolic CLp scaling either_._ For ADE, Supplementary Fig. 1, which is the same as Fig. 1 but stratified on adult ER (i.e., low, intermediate and high ER), shows the wide variability in ‘true’ relative paediatric CLp driven by isoenzyme maturation in each age, leading to both over- and under-predictions of ‘true’ relative paediatric CLp for each ER category. Supplementary Table I also shows that, although PE ranges decrease with increasing ER, every category still includes PE values above 100%.

For hepatic metabolic CLp scaling using AS0.75 + MF_PBPK_, further categorization based on ER of drugs in adults did reveal scenarios for which CLp scaling is systematically accurate. Table IIA shows that PE values for scaled CLp values of drugs with low and intermediate ER lie within a ± 30 and ± 50% range, respectively, when MF_PBPK-liver_ was used for the predictions, except for AAG-bound drugs in neonates of one day. Supplementary Fig. 2 reveals a close agreement between CLp values scaled using AS0.75 + MF_PBPK-liver_ and the ‘true’ relative paediatric CLp for low and intermediate ER drugs, which leads to the acceptable accuracy of CLp scaling in all studied ages, except for drugs binding to AAG in neonates of one day. However, for drugs with a high ER, there are no scenarios based on age and drug properties that systematically lead to accurate CLp scaling with AS0.75 + MF_PBPK-liver_.

**Table II:**
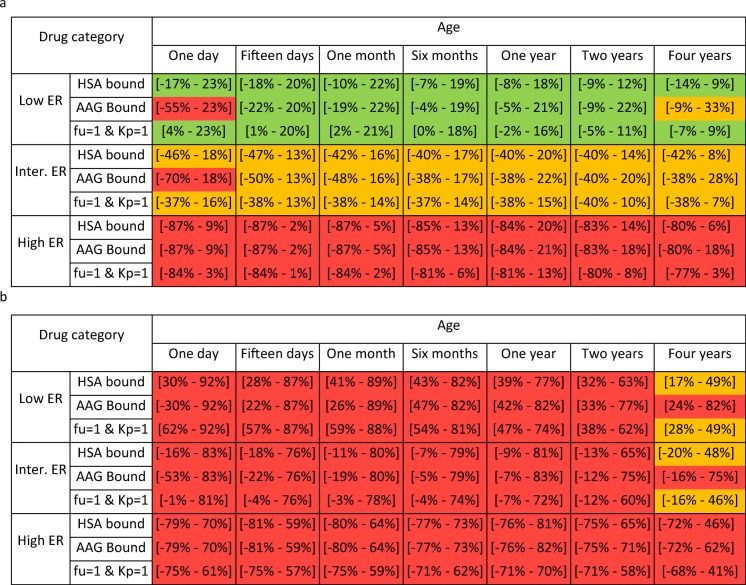
Range of Prediction Errors of CLp Values Obtained When Scaling the CLp of the Hypothetical Drugs Using Either AS0.75 + MF_PBPK-liver_ (A) or AS0.75 + MF_PBPK-microsomes_ (B) for the Investigated Paediatric Ages Categorized per Drug Property

Low, intermediate and high extraction ratios are defined as ER ≤ 0.3, 0.3 < ER ≤ 0.7 and ER > 0.7. fu = 1 and Kp = 1 correspond to drugs not binding to plasma proteins (fu = 1) that are also in equilibrium between plasma and red blood cells (Kp = 1). HSA bound corresponds to drugs that diffuse into red blood cells to different extents and that bind to HSA to different extents (including fu = 1). AAG bound corresponds to drugs that diffuse into red blood cells to different extents and that bind to AAG to different extents (including fu = 1). HSA, human serum albumin; AAG, alpha-1 acid glycoprotein; AS0.75 + MF_PBPK_liver_, AS0.75 in combination with a maturation function expressed in percentage of adult unbound intrinsic clearance per gram of liver; AS0.75 + MF_PBPK_microsomes_, AS0.75 in combination with a maturation function expressed in percentage of adult unbound intrinsic clearance per microgram of microsomes. Colours indicate the PE category, with PE range for all hypothetical drugs lying within ± 30% in green, within ± 50% in orange and including absolute values higher than 50% in red.

Regarding hepatic metabolic CLp scaling using MF_PBPK-microsomes_, Table IIB shows that after additional categorization of the results based on ER, all PE ranges included values outside ± 30% and most of them included PE values outside ± 50% regardless of the drug category. Supplementary Fig. 3 shows a shift in which scaling with this approach moves from predominantly over-estimation of relative paediatric CLp for drugs with a low ER in all ages, towards under-prediction of relative paediatric CLp in all ages with increasing ER of the hypothetical drugs. For this method, no scenarios can however be defined based on age and drug properties that lead to systematically accurate scaling.

## Discussion

As previous analyses have shown that hepatic metabolic CLp scaling based on body weight alone is not systematically accurate in patients younger than 5 years (2,16), the aim of this study was to systematically assess the hepatic metabolic CLp scaling accuracy of ADE and AS0.75 + MF_PBPK_ in children younger than five years. Since this systematic assessment was performed using a PBPK-based simulation workflow analogue to previous analyses of other scaling methods (2,5,16), the reported accuracy of the different methods can be directly compared.

Whereas ADE scaling was found to perform better than standard AS0.75 scaling in all ages, ADE does not systematically lead to accurate scaling of hepatic metabolic CLp from adult to children younger than 5 years. This is due to the significant impact of isoenzyme maturation and drug properties on the ‘true’ relative paediatric CLp, which is not properly accounted for in all cases by ADE. Figure 1 shows that for each age there is not a single exponent that will be able to accurately scale CLp values, as a different exponent will result in a parallel increase or decrease of the horizontal black lines, but it will not be possible to place them such that all relative CLp values are included within those border. This explains the lack of accuracy of scaling methods solely accounting for age and body weight that has been reported for some drugs in young children (1,17). Therefore, although ADE scaling leads to accurate hepatic metabolic CLp scaling for some drugs and isoenzyme maturations in each age, it has not been possible to develop guidelines to *a priori* predict whether this will be the case for a specific individual drug. Using an exponent value of 1.35 in term neonates, as was suggested for drugs metabolized by UGT enzymes (18), may improve scaling of individual drugs in neonates, but it will not improve the systematic accuracy of this scaling method since most drugs are metabolized by several isoenzymes and because similar isoenzyme maturation patterns are found between enzyme families, as for instance, CYP2D6 and CYP2B6 activities having a similar maturation pattern as UGTs in neonates (2).

The wide variations in ‘true’ hepatic metabolic CLp values translate into a wider range in ‘true’ allometric exponents of 0.57 to 2.07 across all ages, compared to the range of 0.8 to 1.2 that we reported earlier for children younger than 5 years (2). The previously reported range in allometric exponent values was derived from scenarios in which size-related changes were considered in the absence of maturation in system-specific parameters. The range reported here corresponds to allometric exponents needed to scale ‘true’ adult CLp values to ‘true’ paediatric CLp values that are impacted by size-related changes as well as by maturational changes in isoenzyme activity, plasma protein concentration and haematocrit.

AS0.75 + MF_PBPK_ is a simplified scaling method that, in addition to scaling based on body weight, includes an age-based PBPK function for enzyme maturation. In our analysis, the same isoenzyme maturation functions were used in the PBPK model and the scaling method; thereby, this maturation function is assumed to be known without bias. This scaling method does not take maturational changes in haematocrit and plasma protein abundance into account, but results show that accounting for isoenzyme maturation is sufficient for accurate hepatic metabolic CLp scaling of drugs with a low or intermediate ER in adults. When isoenzyme maturation is expressed as percentage of adult intrinsic clearance per gram of liver (MF_PBPK-liver_), this method leads to PE of all hypothetical drugs between ± 30 and ± 50% for drugs with a low or intermediate ER in adults, respectively, except for drugs bound to AAG in term neonates of 1 day. This is due to the decreasing variability in relative paediatric CLp with decreasing ER values, because isoenzyme maturation is the main driver of relative paediatric CLp for drugs that have a low or intermediate ER in adults. The lack of accuracy in one day term neonates for drugs binding to AAG is due to the steep increase in AAG concentration in the first days of life, leading to a wide variation in relative paediatric CLp for different hypothetical drugs binding to this plasma protein to varying extents (19). For drugs that have a high ER in adults, the ER decreases in children with decreasing enzyme maturation, and, as a result, the impact of hepatic blood flow on CLp will decrease as well (20). This shift in the contribution of hepatic blood flow is not accounted for in the scaling method. As such, for AAG-bound drugs and for drugs with a high ER in adults, PBPK models are required for accurate CLp scaling from adults to neonates of one day and to children younger than 5 years, respectively.

In scaling hepatic metabolic CLp with the AS0.75 + MF_PBPK_ method, the choice of the PBPK function that is used is of high importance. While both MF_PBPK-liver_ and MF_PBPK-microsomes_ account for isoenzyme maturation, only MF_PBPK-liver_ also accounts for age-related changes in MPPGL (microsomal protein per gram of liver). Expressing isoenzyme maturation as percentage of adult intrinsic clearance per microgram of microsomes (MF_PBPK-microsomes_) leads to inaccurate CLp predictions regardless of drug properties in children under five years of age. Until 2008, MPPGL maturation had not been characterized and, therefore, isoenzyme maturation was expressed as percentage of adult intrinsic clearance per gram of liver (MF_PBPK-liver_) (21). Afterwards, MPPGL maturation was implemented in commercial PBPK software packages, and isoenzyme maturation functions were adapted accordingly to be expressed in percentage of adult intrinsic clearance per microgram of microsomes. As the units of isoenzyme maturation functions are not always reported in literature (22) and because selecting the appropriate MF_PBPK_ is of utmost importance when using AS075 + MF_PBPK_, reporting these units for enzyme maturation functions should be encouraged.

In those cases where after scaling plasma clearance with body weight using a fixed 0.75 allometric exponent, a maturation function is estimated from clinical PK data instead of using enzyme maturation functions as implemented in PBPK models, it is often assumed that the estimated maturation function reflects isoenzyme maturation for drugs undergoing hepatic metabolism. From our results as depicted in Fig. 2, it can be deduced that this is not always the case, as there is only limited overlap between the ‘true’ relative paediatric CLp and the AS0.75 + MF_PBPK_ scaled predictions. The explanation may be that these estimated maturation functions also aggregate the impact of drug properties on clearance maturation that are not properly accounted for. This is in line with previous finding from Strougo *et al.* (4,23).

The application of the PBPK-based framework was an essential part of the current investigation as a clean and systematic evaluation on the impact of individual drug-specific and system-specific parameters is not possible with real data. In a clinical situation, elimination pathways and the impact of changes in individual drug-specific and system-specific parameters cannot be studied in isolation. Moreover, the total number of drugs prescribed in the paediatric population is far too limited to be able to perform a systematic assessment that can support generalizable conclusions for all current and future small molecule drugs. Finally, values of ‘true’ hepatic metabolic CLp from real data are at best approximated by deriving them from observed concentration values that are inevitably obtained with experimental error. The current analysis identifies the theoretical boundaries in PE and ‘true’ allometric exponents for hepatic metabolic CLp between which all current and future small molecular drugs can be predicted to lie *a priori*.

Because isoenzyme maturation was studied as a near continuous variable within the range of reported enzyme maturation values for each age, this analysis covers all possible combinations of hepatic metabolism by multiple isoenzymes contributing to hepatic metabolic CLp to various extents. However, the analysed scenarios do assume the maturation profile of the isoenzymes to be known without bias. For drugs with low or intermediate ER that are metabolized by multiple isoenzymes, scaling hepatic metabolic CLp therefore requires knowledge on the fraction metabolized by each isoenzyme in adults and the MF_PBPK-liver_ of each isoenzyme involved in the drug clearance.

CYP3A7 is an example of isoenzyme often found to be involved in drug metabolism in the paediatric population when other isoenzymes are highly immature. As this isoenzyme is not functionally present in adults, CLp values could not be scaled from adult values based on the maturation profile of this isoenzyme. Although clinically observed total CLp values cannot be directly compared to the hepatic metabolic CLp studied in isolation in the current work, we accounted for the observation that in clinical situations when elimination routes are highly immature other elimination routes take over, by setting a lower limit of 5% isoenzyme maturation.

The scaling accuracy of ADE and AS0.75 + MF_PBPK_ for other elimination routes, including renal excretion, and for scenarios involving multiple elimination mechanisms remains subject of further investigation. Finally, information on maturation of most system-specific parameters in preterm neonates is currently still lacking. Similarly, there is a lack of information on transporter ontogeny in the entire paediatric population. Therefore, further investigation on the systematic accuracy of CLp scaling for all drugs in preterm neonates and for substrates of transporters on hepatocytes in all paediatric ages remains to be performed once the required information for these assessments becomes available.

In conclusion, when scaling CLp from adults to children younger than five years, solely accounting for age and body weight without taking drug properties and enzyme maturation into consideration, will likely not yield systematically accurate scaling for hepatic metabolic CLp. All paediatric CLp values for low and intermediate ER drugs can be scaled with a PE of ± 50% using AS0.75 + MF_PBPK_ except for drugs binding to AAG in neonates of one day, provided the MF_PBPK-liver_ is used thereby accounting for both isoenzyme and MPPGL maturation. For other drugs, no simple scaling method is systematically accurate and their CLp should be scaled using PBPK models.

## Electronic supplementary material

ESM 1 (DOCX 22 kb)

ESM 2 (DOCX 34 kb)

ESM 3 (DOCX 1071 kb)

ESM 4 (DOCX 1229 kb)

ESM 5 (DOCX 1164 kb)

ESM 6 (DOCX 1223 kb)
