# Supplementary material for: Scaling Drug Clearance from Adults to the Young Children for Drugs Undergoing Hepatic Metabolism: A Simulation Study to Search for the Simplest Scaling Method
Source: AAPS J. 2019 Mar 8;21(3):38. doi: 10.1208/s12248-019-0295-0 (PMC6505506; doi:10.1208/s12248-019-0295-0)
Supplement: Supplementary file 1 — (DOCX 22 kb) [file 12248_2019_295_MOESM1_ESM.docx]

***Table S1*** *Range of prediction errors of CLp values obtained when scaling the CLp of the hypothetical drugs using ADE for the investigated paediatric ages categorized per drug property*

| Drug category | | Age | | | | | | |
| --- | --- | --- | --- | --- | --- | --- | --- | --- |
|  |  | One day | Fifteen days | One month | Six months | One year | Two years | Four years |
| Low ER | HSA bound | [-49% - 1224%] | [-47% - 1220%] | [-39% - 1305%] | [-20% - 853%] | [-32% - 701%] | [-38% - 479%] | [-33% - 338%] |
|  | AAG Bound | [-74% - 1224%] | [-50% - 1220%] | [-45% - 1305%] | [-18% - 853%] | [-30% - 739%] | [-37% - 536%] | [-29% - 437%] |
|  | fu=1 & Kp=1 | [-37% - 1205%] | [-37% - 1201%] | [-33% - 1285%] | [-16% - 840%] | [-30% - 690%] | [-37% - 472%] | [-29% - 332%] |
| Inter. ER | HSA bound | [-53% - 968%] | [-51% - 966%] | [-44% - 1036%] | [-26% - 679%] | [-34% - 556%] | [-40% - 377%] | [-35% - 264%] |
|  | AAG Bound | [-72% - 968%] | [-52% - 966%] | [-48% - 1036%] | [-25% - 679%] | [-32% - 583%] | [-39% - 417%] | [-32% - 336%] |
|  | fu=1 & Kp=1 | [-44% - 959%] | [-44% - 956%] | [-41% - 1024%] | [-22% - 670%] | [-31% - 549%] | [-38% - 372%] | [-32% - 260%] |
| High ER | HSA bound | [-56% - 580%] | [-53% - 576%] | [-51% - 621%] | [-33% - 409%] | [-36% - 332%] | [-42% - 217%] | [-37% - 149%] |
|  | AAG Bound | [-69% - 580%] | [-54% - 576%] | [-51% - 621%] | [-33% - 409%] | [-36% - 345%] | [-42% - 239%] | [-36% - 191%] |
|  | fu=1 & Kp=1 | [-52% - 557%] | [-53% - 555%] | [-49% - 597%] | [-29% - 390%] | [-32% - 316%] | [-39% - 206%] | [-35% - 141%] |

Low, intermediate and high extraction ratios are defined as ER ≤ 0.3, 0.3 < ER ≤ 0.7, and ER > 0.7. fu=1 & Kp=1 corresponds to drugs not binding to plasma proteins (fu=1) that are also in equilibrium between plasma and red blood cells (Kp=1). HSA bound corresponds to drugs that diffuse into red blood cells to different extents and that bind to HSA to different extents (including fu=1). AAG bound corresponds to drugs that diffuse into red blood cells to different extents and that bind to AAG to different extents (including fu=1). HSA, human serum albumin; AAG, alpha-1 acid glycoprotein
