# Supplementary material for: Scaling Drug Clearance from Adults to the Young Children for Drugs Undergoing Hepatic Metabolism: A Simulation Study to Search for the Simplest Scaling Method
Source: AAPS J. 2019 Mar 8;21(3):38. doi: 10.1208/s12248-019-0295-0 (PMC6505506; doi:10.1208/s12248-019-0295-0)
Supplement: Supplementary file 2 — (DOCX 34 kb) [file 12248_2019_295_MOESM2_ESM.docx]

**Appendix 1** Methodology

System-specific parameters

PBPK simulations were performed for typical paediatric individuals of various ages and for a typical 25 years old adult. Demographic values (average for males and females) were taken from the CDC growth charts ^1^ for the typical paediatric individuals and from the Simcyp® (Simcyp Ltd, Sheffield, UK) V15.R1 library for a typical adult (see Appendix Table 1). Body surface area (BSA) was estimated using the equations of Dubois and Dubois ^2^ for children weighing >15kg, and of Haycock *et al.* ^3^ for those weighing ≤15kg, as implemented in Simcyp® V15.R1.

PBPK system-specific parameters were taken from the Simcyp® V15.R1 library, including maturation patterns in the hepatic blood flow (Qh), plasma protein concentrations for human serum albumin (HSA) and alpha-1 acid glycoprotein (AAG), haematocrit, liver size, and microsomal protein per gram of liver (MPPGL).

For CL_int,mic_ maturation (MF_PBPK-microsomes_), a realistic range was defined for each paediatric age using the Simcyp® library for all isoenzymes, except for SULT1A1 for which maturity was taken to have been reached at birth ^4^. These MF_PBPK-microsomes_ ranges correspond to the minimum and maximum MF_PBPK-microsomes_ for each investigated paediatric age (see Appendix Table 1), with a minimum limit of 5% being set when minimum reported values were below this limit.

***Appendix Table 1*** *Demographic characteristics of the 8 typical individuals implemented in the PBPK-based simulation workflow and their corresponding system-specific parameters values*

| **Demographic values** | | | | | | | | |
| --- | --- | --- | --- | --- | --- | --- | --- | --- |
| Age | 1 day | 15 days | 1 months | 6 months | 1 year | 2 years | 4 years | 25 years |
| Bodyweight(kg) | 3.45 | 3.69 | 4.30 | 7.55 | 9.90 | 12.35 | 17.10 | 72.65 |
| Height (cm) | 49.75 | 53.40 | 54.25 | 66.00 | 74.75 | 86.00 | 101.98 | 172.30 |
| BSA (m^2^) | 0.22 | 0.24 | 0.26 | 0.38 | 0.46 | 0.55 | 0.69 | 1.86 |
| **System-specific parameters** | | | | | | | | |
| Qh^a^ (L/h) | 6.55 | 7.07 | 7.83 | 12.95 | 17.65 | 24.65 | 36.64 | 87.92 |
| HSA^b^ (g/L) | 35.78 | 36.25 | 39.94 | 42.07 | 42.90 | 43.73 | 41.26 | 43.94 |
| AAG^c^ (g/L) | 0.2678 | 0.5340 | 0.5497 | 0.6774 | 0.7172 | 0.7512 | 0.8406 | 0.6847 |
| Hematocrit (%) | 51.93 | 41.66 | 38.14 | 35.11 | 35.78 | 36.79 | 37.92 | 40.74 |
| Liver size (g) | 133 | 143 | 159 | 249 | 313 | 385 | 501 | 1614 |
| MPPGL^d^ | 25.53 | 25.57 | 25.60 | 25.99 | 26.45 | 27.36 | 29.12 | 39.79 |
| MF_PBPK-microsomes_^e^ | | | | | | | | |
| CYP1A2 ^f^ (%) | 24 | 28 | 35 | 118 | 150 | 164 | 166 | 100 |
| CYP2A6 ^f^ (%) | 2.10^-9^* | 1.10^-2^* | 0.48 | 99 | 100 | 100 | 100 | 100 |
| CYP2B6 ^f^ (%) | 15 | 17 | 19 | 34 | 47 | 62 | 78 | 100 |
| CYP2C8 ^f^ (%) | 38 | 77 | 86 | 97 | 99 | 99 | 100 | 100 |
| CYP2C9 ^f^ (%) | 40 | 68 | 74 | 87 | 90 | 92 | 100 | 100 |
| CYP2C18-19 ^f^ (%) | 30 | 31 | 33 | 84 | 95 | 97 | 98 | 100 |
| CYP2D6 ^f^ (%) | 6 | 32 | 47 | 84 | 91 | 95 | 98 | 100 |
| CYP2E1 ^f^ (%) | 10 | 29 | 37 | 59 | 67 | 74 | 80 | 100 |
| CYP3A4 ^f^ (%) | 11 | 11 | 13 | 48 | 78 | 96 | 103 | 100 |
| UGT1A1 ^f^ (%) | 0.2* | 7 | 23 | 98 | 104 | 100 | 100 | 100 |
| UGT1A4 ^f^ (%) | 74 | 74 | 74 | 74 | 75 | 77 | 80 | 100 |
| UGT1A6 ^f^ (%) | 15 | 23 | 30 | 63 | 76 | 87 | 94 | 100 |
| UGT1A9 ^f^ (%) | 9 | 10 | 12 | 34 | 52 | 71 | 86 | 100 |
| UGT2B7 ^f^ (%) | 8 | 9 | 9 | 11 | 13 | 18 | 27 | 100 |
| SULT1A1 ^g^ (%) | 100 | 100 | 100 | 100 | 100 | 100 | 100 | 100 |
| Studied range (%) | [5 – 100] | [5 – 100] | [5 – 100] | [11 – 118] | [13 – 150] | [18 – 164] | [27 – 166] | NA |

*^a^ Hepatic blood flow*

*^b^ Plasma protein concentrations for human serum albumin*

*^c^ Plasma protein concentrations for alpha-1 acid glycoprotein*

*^d^ Milligram microsomal protein per gram of liver*

*^e^ Isoenzyme maturation expressed as percentage of adult microsomal unbound intrinsic clearance*

*^f^ values obtained from the Simcyp ® V15.R1 library*

*^g^ values obtained from Hines et al.* ^4^

** A lowest value of 5% was used in the simulations*

System and drug specific parameters

To generate PBPK-based CLp values, Qh was taken from Appendix Table 1. Unbound drug fraction in plasma (fu), blood to plasma ratio (B:P) and whole liver unbound intrinsic clearance (CLint) were derived from system-specific parameters in Appendix Table 1 and from the drug-specific parameters defined under hypothetical drugs in the Methodology section of the manuscript.

Fu in adults was taken as a drug property, with different fu values (ranging from 1% to 100%, with 8 equidistant intermediate values) reflecting different affinities for plasma proteins. Adult fu values were scaled to paediatric patients using the relevant plasma concentration of HSA or AAG in adults ([P]adult) and in paediatric patients ([P]paediatric) (see values in Appendix Table 1) according to equation 1. The hypothetical drugs were assumed to exclusively bind to either HSA or AAG.

$\mathrm{fu}_{\mathrm{paediatric}}= \frac{1}{1+\frac{\left( 1-\mathrm{fu}_{\mathrm{adult}} \right)\times{[P]}_{\mathrm{paediatric}}}{{[P]}_{\mathrm{adult}} \times\mathrm{fu}_{\mathrm{adult}}}}$ (1)

B:P was computed based on the defined Kp, and on haematocrit and fu values in the corresponding age, according to equation 2 ^5^.

$B:P=1+ \left[ Hematocrit \times\left( fu\times Kp-1 \right) \right]$ (2)

Kp values of 0.35, 0.8, and values from 1 to 40 with 38 intermediate equidistant values were selected, reflecting different extents of drug diffusion into the red blood cells ^6,7^. Kp was assumed to not change with age.

CLint values were computed according to equation 3.

$CLint=Liver size \times MPPGL \times\mathrm{MF}_{PBPK-microsomes}\times\mathrm{CL}_{int,mic}$ (3)

In this equation, Liver size and MPPGL are system-specific parameters taken from Appendix Table 1, MF_PBPK-microsomes_ is the isoenzyme maturation expressed as percentage of adult microsomal intrinsic clearance for which the range at each age is also defined in Appendix Table 1, and CL_int,mic_ was taken as a drug-specific parameter. As defined under hypothetical drugs in the Methodology section of the manuscript, CL_int,mic_ is the adult unbound intrinsic clearance value of one microgram of liver microsomes, ranging between 0.56·10^-6^ and 0.209·10^-3^ mL.min^-1^.µg^-1^ microsomal protein ^8^, with 98 equidistant intermediate values. These different values reflect difference in both affinities for and abundances of isoenzymes between different drugs.

Dispersion model

‘True’ total hepatic plasma clearance (CLp) values were computed using the dispersion model (Equations 4 to 9). The dispersion model was selected as it has been reported to more accurately predict hepatic CLp than the well-stirred model for highly cleared drugs, while both models lead to equivalent clearance predictions for other drugs ^9^.

$\mathrm{CLp}=\mathrm{CL}_{B}\times B:P$ (4)

$\mathrm{CL}_{B}=Qh\times ER$ (5)

$ER=1-F_{H}$ (6)

$F_{H}= \frac{4a}{{(1+a)}^{2}\exp\left\{ {(a-1)}/{2D_{N}} \right\}-{(1-a)}^{2}\exp\left\{ {-(a+1)}/{2D_{N}} \right\}}$ (7)

$a= {(1+4R_{N}\times D_{N})}^{1/2}$ (8)

$R_{N}=(\mathrm{fu}/{B:P})\times\mathrm{CLint}/\mathrm{Qh}$ (9)

In these equations, $\mathrm{CLp}$ is the overall total (i.e., bound and unbound) hepatic plasma clearance, CL_B_ is the total whole blood clearance, B:P is the blood to plasma ratio, Qh is the hepatic blood flow, ER is the hepatic extraction ratio, fu is the unbound drug fraction in plasma, CLint is the whole liver unbound intrinsic clearance, R_N_ is the efficiency number and D_N_ is the axial dispersion number. For the axial dispersion number (D_N_) a value of 0.17 was used ^10^.

MF_PBPK_ computation.

For the scaling of clearance using AS0.75+ MF_PBPK,_ two different MF_PBPK_ were used:

- MF_PBPK-liver_ which is expressed as percentage of adult unbound intrinsic clearance per gram of liver and accounts for maturation in both isoenzyme activity and MPPGL. MF_PBPK-liver_ was computed as the product of MPPGL maturation (i.e., the paediatric to adult MPPGL ratio) and the isoenzyme maturation expressed as percentage of adult microsomal intrinsic clearance (i.e., $\mathrm{MF}_{PBPK-microsomes}$) according to equation 10.

$\mathrm{MF}_{PBPK-liver}=\frac{\mathrm{MPPGL}_{\mathrm{paediatric}}}{\mathrm{MPPGL}_{\mathrm{adult}}}\times\mathrm{MF}_{PBPK-microsomes}$ (10)

- MF_PBPK-microsomes_ is expressed as percentage of adult unbound intrinsic clearance per microgram of microsomes and only accounts for maturation of isoenzyme activity.

**References**

1. Centers for Disease Control and Prevention NCHS. Length-for-age and weight-for-age percentiles. (2000).

2. Du Bois, D. & Du Bois, E. F. A formula to estimate the approximate surface area if height and weight be known. 1916. *Nutrition* **5**, 303-11; discussion 312–3

3. Haycock, G. B., Schwartz, G. J. & Wisotsky, D. H. Geometric method for measuring body surface area: a height-weight formula validated in infants, children, and adults. *J. Pediatr.* **93**, 62–6 (1978).

4. Hines, R. N. The ontogeny of drug metabolism enzymes and implications for adverse drug events. *Pharmacol. Ther.* **118**, 250–67 (2008).

5. Maharaj, A. R., Barrett, J. S. & Edginton, A. N. A workflow example of PBPK modeling to support pediatric research and development: case study with lorazepam. *AAPS J.* **15**, 455–64 (2013).

6. Uchimura, T., Kato, M., Saito, T. & Kinoshita, H. Prediction of human blood-to-plasma drug concentration ratio. *Biopharm. Drug Dispos.* **31**, n/a-n/a (2010).

7. Hinderling, P. H. Red blood cells: a neglected compartment in pharmacokinetics and pharmacodynamics. *Pharmacol. Rev.* **49**, 279–95 (1997).

8. Nikolic, K. & Agababa, D. Prediction of hepatic microsomal intrinsic clearance and human clearance values for drugs. *J. Mol. Graph. Model.* **28**, 245–52 (2009).

9. Ridgway, D., Tuszynski, J. A. & Tam, Y. K. Reassessing models of hepatic extraction. *J. Biol. Phys.* **29**, 1–21 (2003).

10. Naritomi, Y. *et al.* Prediction of human hepatic clearance from in vivo animal experiments and in vitro metabolic studies with liver microsomes from animals and humans. *Drug Metab. Dispos.* **29**, 1316–24 (2001).
