# Supplementary material for: Scaling Drug Clearance from Adults to the Young Children for Drugs Undergoing Hepatic Metabolism: A Simulation Study to Search for the Simplest Scaling Method
Source: AAPS J. 2019 Mar 8;21(3):38. doi: 10.1208/s12248-019-0295-0 (PMC6505506; doi:10.1208/s12248-019-0295-0)
Supplement: Supplementary file 5 — (DOCX 1164 kb) [file 12248_2019_295_MOESM5_ESM.docx]

**Supplementary Figure 3**


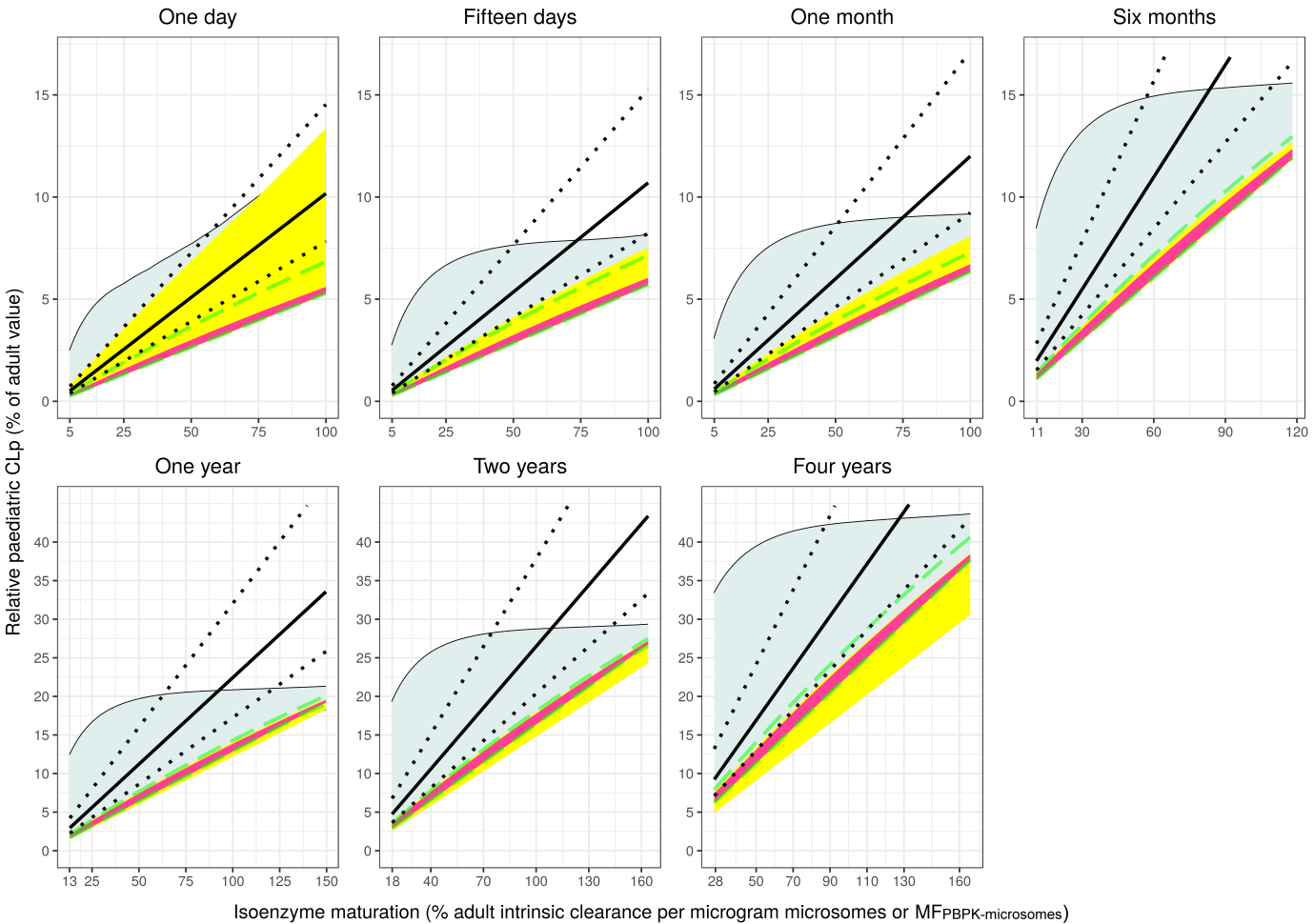


A


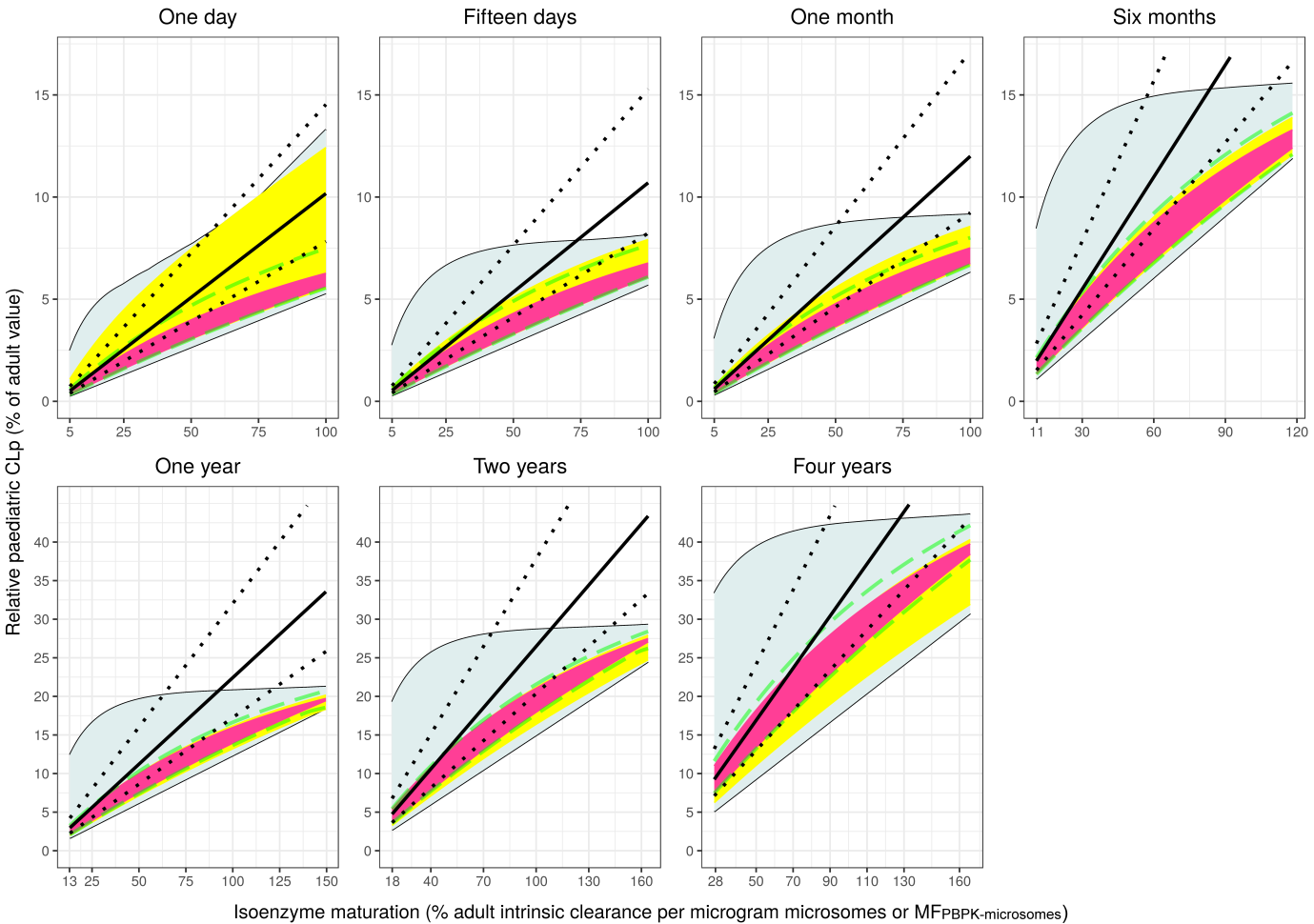


B


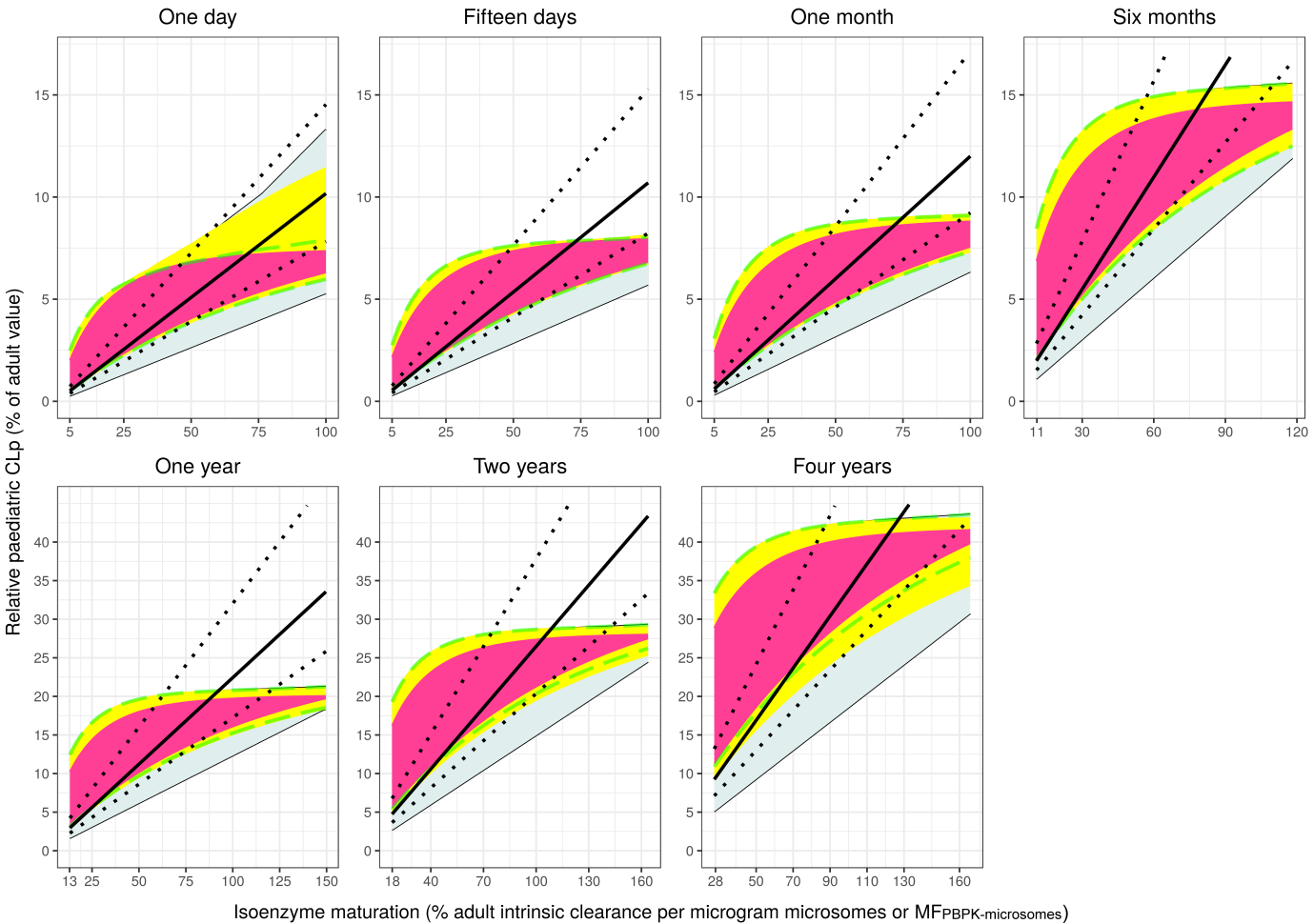


C

***Supplementary Figure 3*** *Relative paediatric CLp (% of adult value) obtained with AS0.75+MF_PBPK-microsomes_ scaling (solid black line with ± 30% PE as dotted black lines) and ‘true’ relative paediatric CLp (pink, green and yellow areas) for all hypothetical drugs versus the respective isoenzyme maturation range in the studied typical paediatric individuals. Drugs are categorized by extraction ratio (ER) in adults with low ER (≤ 0.3) in panel A, intermediate ER (0.3-0.7) in panel B, and high ER (> 0.7) in panel C. Different colours represent hypothetical drugs with different properties, with the pink shaded area representing drugs within the designated ER category not binding to plasma proteins (fu=1) that are also in equilibrium between plasma and red blood cells (Kp=1). The area delimited by the two green dashed lines and the yellow shaded area are used to depict drugs within the designated ER category that diffuse into red blood cells to different extents and that bind to HSA or AAG, respectively, to different extents (including fu=1). Under the pink area, the pink and yellow areas overlap completely, therefore the combination of pink and yellow areas shows the results for drugs within the designated ER category binding to AAG. The blue shaded area depicts the range for the remaining drugs with ER values outside the designated ER category in each plot. Note that the scales on the x- and y-axes may be different for different ages.*
